# A bibliometric analysis of clinical study literature of traditional Chinese medicine therapies for smoking cessation

**DOI:** 10.18332/tid/86330

**Published:** 2018-04-20

**Authors:** Jian-Hua Wang, Mei Wang, Shu-Chun Liu, Xiao-Feng Du, Mei Han, Jun-Feng Liu, Wei Qin, Bin Chen, Robbert van Haselen, Jian-Ping Liu

**Affiliations:** 1 Science and Technology Department, Liaoning University of Traditional Chinese Medicine, Shenyang 110847, China; 2 School of Preclinical Medicine, Liaoning University of Traditional Chinese Medicine, Shenyang 110847, China; 3 Medical Library, Liaoning University of Traditional Chinese Medicine, Shenyang 110847, China; 4 Centre for Evidence-Based Chinese Medicine, Beijing University of Traditional Chinese Medicine, 11 Bei San Huan Dong Lu, Chaoyang District, Beijing 100029, China; 5 International Forum on Integrative Medicine

**Keywords:** bibliometric analysis, clinical studies, smoking cessation, traditional Chinese medicine therapy

## Abstract

**INTRODUCTION:**

Traditional Chinese medicine (TCM) is commonly used for smoking cessation in China. The aim of this study is to perform a comprehensive literature search to identify clinical studies on TCM therapies for smoking cessation.

**METHODS:**

Publications of randomized controlled trials, controlled clinical studies, cohort studies, case-control studies, case series and case reports, reviews and cross-sectional studies on smoking cessation using TCM therapies were retrieved from seven databases from their inception to February 2017. The following data were extracted and analyzed: study type, year of publication, language, country or region, journals, participants, intervention and comparison, and outcome.

**RESULTS:**

In total, 260 publications on TCM therapies for smoking cessation were identified from 1980 to 2016, including 52 randomized clinical trials, 7 controlled clinical studies, 1 cohort study, 110 case series, 18 case reports, 50 narrative reviews, 17 systematic reviews, and 5 cross-sectional studies. Of these, 68.5% (178) were published in Chinese and the remaining published in English. Mainland China (n=129, 49.6%) was the leading country in this field, followed by USA (n=27, 10.4%) and UK (n=25, 9.6%). A total of 36 645 participants from 40 countries with age ranging from 12 to 86 years were involved in 188 clinical studies (excluding reviews and cross-sectional studies). The most commonly reported therapies were auricular acupressure (25, 13.3%), body acupuncture (14, 7.4%), and body acupuncture plus auricular acupressure (14, 7.4%). Composite outcomes were most frequently reported (110, 58.5%).

**CONCLUSIONS:**

A substantial number of clinical studies have been conducted and published on TCM therapy for smoking cessation, mainly focusing on acupuncture stimulation techniques. The findings suggest that future research should pay more attention to acupuncture for smoking cessation.

## INTRODUCTION

Smoke from cigarettes contains a variety of harmful substances^1-3^ and smoking is one of the principal causes of chronic obstructive pulmonary disease, cancer, atherosclerotic cardiovascular disease and cerebrovascular disease^4,5^, which are the most common causes of morbidity and mortality^6-8^. The World Health Organization (WHO) reports that tobacco-use causes the death of about 6 million people worldwide every year^9^. More than 5 million of these deaths are associated directly with the use of tobacco, with more than 600 000 of these deaths corresponding to non-smokers exposed to secondhand smoke^9^. Smoking addiction is a complex problem, involving physical, psychological, social and other factors^10^, mainly due to nicotine, the psychoactive substance contained in cigarettes^11,12^. The development of smoking-related diseases and the associated risk of death significantly decrease in smokers who quit smoking^13,14^. Under the guidance of WHO, countries worldwide are taking active measures against smoking^15,16^.

The most common recommended approaches to quitting smoking include nicotine replacement therapy, antidepressants and psychological support^17-29^. Despite the high cost, the effectiveness of these approaches is limited and side effects (such as anorexia, nausea, constipation, headache, drowsiness or insomnia) can occur^30,31^. Therefore, new approaches that could help smokers to quit smoking are warranted. Traditional Chinese Medicine (TCM) therapy is widely used to aid smoking cessation, and because of its relative safety, low cost and claimed effectiveness, it has attracted increasing attention worldwide^32,33^. The application of TCM therapy to smoking cessation has a long history in China. Its aim is to inhibit the craving for cigarettes and to alleviate withdrawal symptoms^34^. There is widespread interest in TCM therapy for smoking cessation, especially the use of acupuncture which has been investigated in a series of clinical studies. Despite this, the effectiveness of TCM therapy for smoking cessation is still controversial.

We are, therefore, committed to obtaining insights on the role of TCM therapy in the field of smoking cessation. This comprehensive bibliometric analysis aims to provide an overview of the status of TCM therapy as an intervention for smoking cessation worldwide. We intend to identify, develop and offer appropriate evidence-based TCM approaches to help people to quit smoking, as well as to provide a framework for further evaluating the efficacy of the most promising TCM approaches for smoking cessation.

## METHODS

### Search strategy

We searched five Chinese and two English language electronic databases (see Additional file 1 for details). With two library staff members (SCL and JFL), we developed the search terms and conducted literature searches. In order not to miss relevant articles, we decided not to set other limitations on the searching.

### Eligibility criteria

We included all types of clinical studies: randomized controlled trials (RCTs), clinical controlled trials (CCTs), cohort studies, case-control studies, case series and case reports. Descriptive epidemiological studies (cross-sectional surveys), systematic reviews and traditional reviews on TCM therapies for smoking cessation were also included. Editorials and letters to the editor were excluded. When the same data were published more than once, we chose those that provided more comprehensive information. We considered documents to be eligible those which aimed at evaluating the therapeutic effect or safety of smoking cessation by TCM therapies. Those studies that were conducted to study mechanisms of action were not included.

### Types of interventions

For clinical studies, we placed no limitations on the types of TCM therapies, such as acupuncture, acupressure, massage, herbal formulae, herbal extracts, plaster on acupuncture points, qi gong, Tai chi, and so on. In fact, we intended to understand the overall view of the therapeutic effect of all types of TCM therapies in the field of smoking cessation. Monotherapy (acupuncture, qi gong, etc.) and multiple therapies that combined TCM therapy with other interventions (acupuncture plus auricular acupressure, acupuncture plus nicotine replacement therapy, etc.) were all included.

### Comparisons

For controlled clinical studies, including RCTs, CCTs, cohort studies and case-control studies, the control interventions were: no treatment, sham treatment, waiting list, other treatment, and combined therapy. The control intervention of some studies was a no-treatment control^35^ or waiting list^36^. Some studies utilized sham acupuncture or placebo as the control intervention^37,38^. Single and combined therapies, such as nicotine replacement therapy^39^, behavioral therapy^36^, TCM therapy plus counseling^40^, and so on, are also often applied as control interventions.

### Data extraction and analysis

One author (XFD) imported the retrieved bibliography citations into NoteExpress (3.2.0.6976), and duplicates were deleted. JHW first screened the articles that clearly did not meet the inclusion criteria based on the reading of titles and abstracts. Next, two review authors (JHW and MH) selected eligible articles by reading the full text independently. JPL was responsible for resolving differences and contradictions in the screening process of the two authors, and for checking the eligibility of included articles. Three review authors (JHW, WQ, BC) extracted data from the included studies with a self-developed electronic data extraction form using the public domain software package Epidata (version 3.1). In case of disagreement, a consensus was reached by discussion with JPL. The extracted variables included: publication characteristics, study design, demographic characteristics, intervention details, control treatment details, and clinical outcomes.

The extracted data were imported into SPSS (release 20.0, IBM, Armonk, NY, USA) and Microsoft Excel (version 12.3.5, Microsoft, Redmond, WA, USA), which were used for the statistical analysis and to describe the characteristics of the data. Categorical variables were represented by frequencies and percentages, while continuous variables were reported as means and standard deviations.

## RESULTS

### General description of included literature

**Fig 1:**
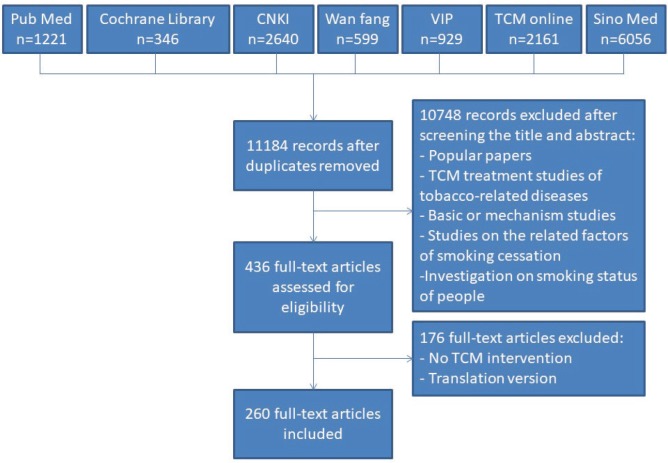
The flowchart of including and existing studies

A total of 260 records were included and analyzed in this study (Figure 1), of these 178 (68.5%) articles were published in Chinese, while 82 (31.5%) articles were in English. Journal articles (244, 93.8%) were the most frequent publication type, followed by dissertations (8, 3.1%), and conference full text papers (6, 2.3%). Most of the studies were conducted by universities or medical colleges (106, 40.8%). Hospitals (96, 36.9%) also played an important role in studies related to smoking cessation using TCM therapy, followed by research institutes (30, 11.5%) and associations (4, 1.9%). Other clinical settings (16, 6.2%) in which smoking cessation studies were carried out included: clinics, community health centers, nursing homes, companies, and foreign aid medical teams. Seven (2.7%) articles provided no information about the institution. Among the 260 articles, 205 (78.8%) studies did not report whether they had been funded, 52 (20%) studies received financial support, and 3 (1.2%) did not have any financial aid.

**Fig 2:**
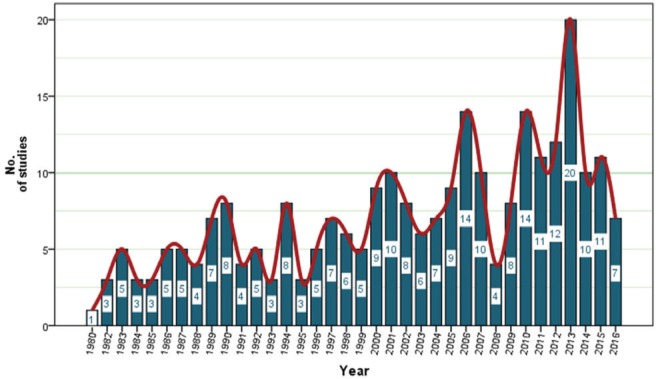
Annual number of publications for smoking cessation using TCM therapy

Annual publications on smoking cessation using TCM therapy are summarized in Figure 2. The earliest publication year was 1980, at that time there was just one article produced. The first study was a case series conducted by a Chinese doctor in West African Senegal. From 1980 to 2016, the annual number of publications related to smoking cessation using TCM therapy increased slightly, with a peak of 14 in 2006. In terms of the number of publications, there was a significant decline until 2008. Since 2009 and 2010, there has been a large increase in publications, peaking in 2013. From 2013 to 2016, the annual number of publications decreased significantly. The long-term trend is a gradual increase in the number of publications.

The top seven countries, in which studies were carried out, were mainland China (129, 49.6%), USA (27, 10.4%), UK (25, 9.6%), Republic of Korea (9, 3.5%), Taiwan, China (7, 2.7%), Australia (5, 1.9%), Malta (4, 1.5%), followed by 31 other countries.

Nearly half of the 260 articles were performed in mainland China, and another 40 studies performed in other countries were conducted by Chinese who were providing medical aid abroad, including twelve African countries, Austria, Germany, Republic of Korea, Kuwait, Lao People’s Democratic Republic, Portugal, Russian Federation, Singapore, Switzerland, Thailand, Turkey, UAE, UK and the USA. Only one study was a collaboration conducted in mainland China and Taiwan, China (Table 1).

**Table 1:** Distribution of countries where the studies of smoking cessation using TCM therapies were performed

*Country*	*Frequency*	*Percentage % N=260*
Mainland China	129	49.6
USA	27	10.4
UK	26	10.0
Republic of Korea	9	3.5
Taiwan, China	7	2.7
Australia	5	1.9
Malta	4	1.5
France	3	1.2
Germany	3	1.2
Hong Kong, China	3	1.2
Singapore	3	1.2
Switzerland	3	1.2
Turkey	3	1.2
No record	3	1.2
Russian Federation	2	0.8
Namibia	2	0.8
Thailand	2	0.8
UAE	2	0.8
Niger	2	0.8
Senegal	2	0.8
Arabia	1	0.4
Austria	1	0.4
Brazil	1	0.4
Canada	1	0.4
Central African Republic	1	0.4
United Republic of Tanzania	1	0.4
Italy	1	0.4
Kuwait	1	0.4
Lao People's Democratic Republic	1	0.4
Netherlands	1	0.4
Morocco	1	0.4
Norway	1	0.4
Portugal	1	0.4
Mauritius	1	0.4
Taiwan, China and Mainland China	1	0.4
Burkina Faso	1	0.4
Mali	1	0.4
Nigeria	1	0.4
Nigeria and Egypt	1	0.4
Togo	1	0.4
Total	260	100.0

Active authors in publishing articles about smoking cessation using TCM therapy are shown in Table 2, with White being the most prolific contributor with 10 articles. Ten active authors were Chinese, and the other active authors were from Republic of Korea, France, USA and Taiwan, China.

**Table 2:** First authors who produced two or more articles on smoking cessation using TCM therapies

*First author*	*Article frequency*	*Percentage % N=260*	*Country*
White AR	10	3.8	UK
Huang JM	3	1.2	Mainland China
Wang YY	3	1.2	Mainland China
Chae Y	2	0.8	Republic of Korea
Clavel F	2	0.8	France
Sood A	2	0.8	USA
Yeh ML	2	0.8	Taiwan, China
Cui M	2	0.8	Mainland China
Fang YA	2	0.8	Mainland China
Gu ZR	2	0.8	Mainland China
Lei ZQ	2	0.8	Mainland China
Li HB	2	0.8	Mainland China
Liu C	2	0.8	Mainland China
Wang XJ	2	0.8	Mainland China
Wang YX	2	0.8	Mainland China
Total	40	15.4	

Table 3 ranks the journals by the number of publications. Chinese Acupuncture & Moxibustion outranked other journals with 21 publications. Of the top 10 active journals, 7 were Chinese journals, and 3 were in English.

**Table 3:** Top ten most active journals in the field of smoking cessation applying TCM therapy

*Journal*	*Frequency*	*Percentage % N=260*	*Language*
Chinese Acupuncture & Moxibustion	21	8.1	Chinese
Shaanxi Journal of Traditional Chinese Medicine	7	2.7	Chinese
Journal of Clinical Acupuncture and Moxibustion	7	2.7	Chinese
Shanghai Journal of Acupuncture and Moxibustion	6	2.3	Chinese
Journal of Traditional Chinese Medicine	6	2.3	Chinese
Am J Chin Med	5	1.9	English
Evid Based Complement Alternat Med	5	1.9	English
J Altern Complement Med	5	1.9	English
Journal of Liaoning University of Traditional Chinese Medicine	5	1.9	Chinese
Journal of Sichuan of Traditional Chinese Medicine	5	1.9	Chinese
Total	72	27.7%	

### Study types

**Fig 3:**
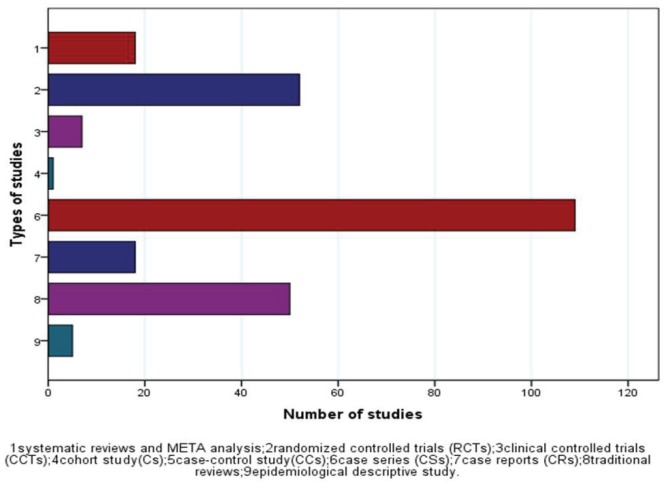
The distribution of study types

Figure 3 illustrates that the great majority of the literature consists of case series (110, 42.3%), followed by RCTs (52, 20.0%), traditional reviews (50, 19.2%), individual case reports (18, 6.9%), systematic reviews (17, 6.5%) and CCTs (7, 2.7%). Cross-sectional descriptive epidemiological studies (5, 1.9%) accounted for less of the data and cohort studies were the least common (1, 0.3%).

Case series was the earliest type of study^41^, which was reported in the Journal of Traditional Chinese Medicine, in Chinese, with smoking cessation rate reaching 90.95% after intervention by body acupuncture and auxiliary ear acupoint buried needle. Case series was also the most frequently applied study type for investigating the effectiveness of TCM for smoking cessation, and it was particularly popular in China. Of the case series, 103 were conducted by Chinese, while only 7 were conducted by other countries. Compared to RCTs, case series are easier to complete. The first RCTs^42^ about smoking cessation using TCM therapy were published in Am J Chin Med in 1982, conducted in the USA comparing the smoking cessation efficacy of real ear acupuncture (RA) and sham ear acupuncture. Most of the systematic reviews (16/17) were published in English journals. The British Journal of General Practice reported the first meta analysis of studies on the effect of acupuncture on addiction^43^.

### Participants

There were 36 645 participants in the 188 clinical studies, excluding the 67 reviews and 5 descriptive epidemiological studies. The largest sample size was 8803 (a case series conducted in Guangxi China with a reported rate of smoking cessation of 95.0%, while the smallest study had an N=1 (case report). In all, 21 990 (60.0%) were male and 14 655 (40.0%) were female. Of the 188 clinical studies, only 11 studies reported a detailed ethnic distribution of participants. The analysis of extracted data indicated that the youngest participant was 12 years old and the oldest 86 years. The duration of regular active or passive smoking of all participants varied from 3 months to 70 years. There was a wide variation in the number of cigarettes consumed daily (3-100).

### Therapy category

A total of 173 (92.0%) publications of 188 clinical studies did not report any information about diagnostic criteria of tobacco dependence. Only 22 (11.7%) studies reported the use of syndrome differentiation to guide treatment. Regarding the treatment principle of traditional Chinese medicine, 34 (18.1%) clinical studies described it in detail, including the treatment principle of Tiao Li Fei Qi, Shu Gan Jian Pi, Zhen Jing An Shen. Overall, 142 (75.5%) of the 188 studies clearly recorded the operational steps and specifications of the treatment approach. Details on the course of treatment could not be found in 9 (4.8%) articles, and another 11 (5.9%) articles lacked detail on the treatment sessions. In the remaining 168 studies, the average duration of treatment varied from 10 minutes to 12 weeks. Table 4 illustrates the varied nature of TCM interventions employed to aid smoking cessation.

**Table 4:** Number of different categories of therapies in clinical studies

*Categories*	*Intervention*	*Frequency*
Single treatment	auricular acupressure	25
	body acupuncture	14
	auricular acupuncture	5
	embedded needling at auricular acupoint	5
	qigong	5
	auricular electroacupuncture	4
	auricular acupoint stimulation using lasers	4
	herb formula	4
	Chinese herb extracts	4
	auricular acupoint stimulation using electricity	2
	acupoint stimulation using lasers	2
	acupoint application	2
	herb tea	2
	body electroacupuncture	1
	scalp acupuncture	1
	Dai Zhen Gao	1
	acupoint stimulation using electricity	1
	acupoint embedded line	1
	acupuncture and moxibustion	1
	tea filters	1
	treatment at Tian Mei acupoint	1
	standing pile	1
	Inedia	1
Two types of treatment combined	body acupuncture + auricular acupressure	14
	body acupuncture + embedded needling at auricular acupoint	5
	auricular acupressure + psychological intervention	4
	body electroacupuncture + auricular acupressure	4
	body acupuncture + auricular acupuncture	3
	auricular acupuncture + auricular acupressure	3
	body acupressure + auricular acupressure	2
	auricular electroacupuncture + embedded needling at	
	auricular acupoint	2
	auricular electroacupuncture + auricular acupressure	2
	auricular acupressure + multimedia instruction	2
	body acupuncture + moxibustion	1
	body acupuncture + body electroacupuncture	1
	body acupuncture + bloodletting	1
	body acupuncture + scalp acupuncture	1
	body acupuncture + wrist-ankle acupuncture	1
	body acupuncture + smoking therapy	1
	body acupuncture + psychological intervention	1
	body acupuncture + body acupressure	1
	body electroacupuncture + cupping	1
	body electroacupuncture + embedded needling at body acupoint	1
	body acupoint massage + auricular acupoint massage	1
	auricular acupuncture + embedded needling at auricular acupoint	1
	auricular acupuncture + the Internet-assisted smoking cessation program	1
	auricular acupressure + auricular acupoint massage	1
	auricular acupressure + scraping	1
	auricular acupressure + body acupoint massage	1
	auricular acupressure + body acupressure	1
	auricular acupressure + decreasing smoke consumption method gradually	1
	embedded needling at auricular acupoint + herb formula	1
	auricular acupoint stimulation using lasers + body acupoint stimulation	
	using lasers	1
	scalp acupuncture + eye acupuncture	1
	Acupoint application + psychological intervention	1
	Acupoint application + varenicline tartrate tables	1
	Chinese herb extracts + smoking cessation message	1
	Chinese herb extracts + behavioral intervention	1
Three types of treatment combined	body acupuncture + herb formula + line point moxibustion of Zhuang medicine	3
	body electroacupuncture + auricular acupuncture + auricular acupressure	2
	body acupuncture + auricular acupuncture + embedded needling	
	at auricular acupoint	2
	body acupuncture + body acupoint massage + auricular acupressure	1
	body acupuncture + auricular acupressure + herb tea	1
	body acupuncture + embedded needling at auricular acupoint + acupoint stimulation using lasers	1
	body acupuncture + embedded needling at auricular acupoint + herb tea	1
	body acupuncture + auricular acupuncture + auricular acupressure	1
	body acupuncture + auricular acupuncture + nicotine replacement therapy (NRT)	1
	body acupuncture + auricular acupuncture + scalp acupuncture	1
	body acupuncture + bloodletting + decreasing smoke consumption method gradually	1
	body acupuncture + manipulation + psychological intervention	1
	body acupuncture + auricular electroacupuncture + auricular acupressure	1
	body electroacupuncture + auricular acupressure + body acupuncture	1
	auricular acupressure + NRT + team support	1
	auricular acupressure + herb cigarette + basic management intervention	1
	auricular acupressure + acupoint application + herb tea	1
	auricular acupuncture + body acupuncture + smoking cessation education	1
	scalp acupuncture + nasal acupuncture + NRT	1
	plum-blossom needling + ear acupressure + acupoint injection	1
Four types of treatment combined	body acupuncture + auricular acupressure + auricular electroacupuncture + Chinese patent medicine	1
	body acupuncture + auricular acupressure + herb tea + psychological intervention	1
	body acupuncture + auricular acupressure + auricular acupuncture + herb tea	1
	body acupuncture + auricular acupressure + body electroacupuncture + psychological intervention	1
More than four types of treatment combined	auricular acupressure + western medicine + psychological intervention + health education + administrative interference + multimedia	1
	acupuncture + individualized counseling + nicotine replacement therapy + stress management + behavior modification	1
	auricular acupoint stimulation using electricity + auricular acupoint stimulation using lasers + nasal acupoint stimulation using electricity + nasal acupoint stimulation using lasers + standardized consulting	1
	the Zhuang medicine comprehensive treatment of acupuncture treatment	1

### Outcomes

Of the 188 clinical studies, 111 (59.0%) reported follow-up after the therapy ranging from 26 hours^44^ to 5 years^45^. Seventy (37.2%) clinical studies failed to report the main efficacy criteria. The TCM therapy effectiveness was reported comprehensively in 111 (59.0%) studies, smoking cessation rate was reported in 77 (41.0%), smoking craving in 24 (12.8%), nicotine dependence scale in 19 (10.1%), changes in withdrawal symptoms in 33 (17.6%), decrease in daily cigarette consumption in 47 (25.0%), change in the taste of cigarettes in 25 (13.3%), and laboratory parameters in 33 (17.6%). Five (2.7%) studies included assessment of quality of life. Forty-one (21.8%) studies focused on the safety of the therapy, including adverse events and/or adverse drug reactions. Two studies (1.1%) reported health economic data.

## DISCUSSION

The present study identified and comprehensively analyzed 260 publications of TCM therapy for smoking cessation. Mainland China was the main country that investigated TCM for smoking cessation. On the other hand, our study also showed that TCM therapy is widely accepted and used in the field of smoking treatment throughout the world. However, international collaboration on this topic is rare. Systematic reviews are important in the field of efficacy evaluation. A systematic review of clinical trials^46^ published in 2014 concluded that there was not enough evidence indicating acupuncture was effective in smoking cessation. This finding is inconsistent with a large body of outcome data on clinical practice.

As a bibliometric study, we tried to identify all available clinical evidence on TCM therapies for smoking cessation. Therefore, all types of experimental and observational studies, review articles and descriptive epidemiological studies related to smoking cessation applying TCM therapy were retrieved. We found a predominance of case series. The number of RCTs, considered by many to be the gold standard for clinical efficacy, was less than half the number of case series. This may be one of the reasons why the efficacy of TCM therapy for smoking cessation is not widely recognized globally. Targeting the individual treatment characteristics and current status of clinical research in TCM, a proposal for evidence grading that focused not only on RCTs but also on cohort studies, case-control studies, case series was suggested^47^, in order to reflect the efficacy of TCM more objectively and correctly.

Due to the diversity and flexibility of TCM therapy, smoking cessation therapy was applied in different ways according to the actual situation of the smokers, the stage of the smoking cessation process^48^ and clinical experience of the researchers. This included single TCM therapies, as well as various treatment combinations, including those with western therapy such as nicotine replacement therapy. The application of TCM syndrome differentiation made the treatment more complicated. It is difficult to develop a unified, repeatable TCM therapy for smoking cessation. This undoubtedly hinders the spread of TCM therapy for smoking cessation and makes it difficult to carry out a systematic review on the beneficial effect of smoking cessation using TCM therapy. An additional table file shows this in more detail [see Additional file 2].

This bibliometric analysis has several limitations, including selection of databases and language restrictions. At present, there is a variety of databases, specialized or generalized. Due to resource constraints, we could not retrieve all the databases, especially some English databases, such as Google Scholar, and the acupuncture academic professional database, which may have led to missing publications. Moreover, limiting our analysis to articles published in Chinese and English, means that we missed publications in other languages, such as French, Korean, Japanese, German, Russian, etc.

## CONCLUSIONS

To our knowledge, the available data related on TCM therapy for smoking cessation has been comprehensively described for the first time based on a systematic bibliometric approach. Our study revealed the characteristics of the literature on TCM therapy for smoking cessation. It included a general description of the available literature, study types, interventions, participants and outcome assessments. This provides a useful overview for researchers and clinicians with an interest in the topic of smoking cessation. The design types of research used in the area of smoking cessation using TCM may provide a reference for future research on evidence evaluation on this topic. The analysis of all kinds of interventions, especially acupuncture, may provide a reference for the selection of interventions for clinical practice or future research of addiction.

Whilst mainland China is the leading country to conduct research on TCM therapy, many other countries are also active in this field. Especially, acupuncture therapy has been applied to help smokers quit smoking worldwide. Despite the importance of smoking cessation using TCM therapy, the extent of international collaboration on this topic is very small. This paper may help to foster further cooperation.

## CONFLICTS OF INTEREST

Each of the authors has completed and submitted an ICMJE form for disclosure of potential conflicts of interest. The authors declare that they have no competing interests, financial or otherwise, related to the current work.
