# Supplementary material for: A bibliometric analysis of clinical study literature of traditional Chinese medicine therapies for smoking cessation
Source: Tob Induc Dis. 2018 Apr 20;16:15. doi: 10.18332/tid/86330 (PMC6659472; doi:10.18332/tid/86330)
Supplement: Supplementary file 1 [file TID-16-15-s1.pdf]

Additional file 1
Literature retrieval strategy

indicators	details
databases	China National Knowledge Infrastructure (CNKI) (1979-02.2017), Chinese Biomedical Database (SinoMed) (1978-02.2017), Chinese Scientific Journal Database (VIP) (1989-02.2017), Wanfang Database (1990-02.2017), Traditional Chinese Medicine online (TCM online) (1949-02.2017), PubMed (1966-02.2017), and the Cochrane Library (1999-02.2017)
retrieval time	from inception to February 2017
MeSH term	”Chinese Herbal” or ”TCM THERAPY” or ” Acupuncture” or ”Tai Ji” and ”Smoking Cessation” or ”Tobacco Use Disorder” or ”Substance Withdrawal Syndrome”
key words	‘‘Chinese medicine’’ or ‘‘integrated traditional Chinese and western medicine’’ or ‘‘Chinese herbs’’ or ‘‘Chinese patent medicine’’ and ‘‘acupuncture’’ or ‘‘electroacupuncture’’ and ‘‘auricular acupuncture’’ or ‘‘laser needle’’ or ‘‘scalp acupuncture’’ or ‘‘needle-embedding’’ or ‘‘Dai Zhen Gao’’ and ‘‘auricular point sticking’’ or ‘‘auricular-plaster’’ or ‘‘acupressure’’ or ‘‘moxibustion’’ or ‘‘manipulation’’ or ‘‘massage’’ or ‘‘cupping’’ or ‘‘hot compress’’ or ‘‘qi gong’’ or ‘‘Tai chi’’ and ‘‘smoking cessation’’ or ‘‘tobacco cessation’’ or ‘‘quit smoking’’ or ‘‘stop smoking’’ or ‘‘preventing smoking’’ or ‘‘withdrawal symptoms’’ or ‘‘withdrawal syndrome’’ or ‘‘nicotine dependence’’
language	The language of publications were limited in Chinese and English
