# Supplementary material for: A bibliometric analysis of clinical study literature of traditional Chinese medicine therapies for smoking cessation
Source: Tob Induc Dis. 2018 Apr 20;16:15. doi: 10.18332/tid/86330 (PMC6659472; doi:10.18332/tid/86330)
Supplement: Supplementary file 2 [file TID-16-15-s2.pdf]

Additional file 2
The profile of 17 system overviews

ID	title	journal	number of studies	result
Liu C 2015[49]	Condition and effectiveness evaluation of acupuncture for smoking cessation	Chinese Acupuncture & Moxibustion	24	Compared with other treatment, acupuncture has positive advantages on short-time abstinence rate, however, it could not increase the long-time abstinence rate.
Ter Riet G 1990[43]	A meta-analysis of studies into the effect of acupuncture on addiction	Br J Gen Pract	15	For smoking cessation, the number of studies with negative outcomes exceeded by far the number with positive outcomes.
Tahiri M 2012[50]	Alternative smoking cessation aids: a meta-analysis of randomized controlled trials	Am J Med	6	Acupuncture and hypnotherapy may help smokers quit.
Ashenden R 1997[51]	A meta-analysis of the effectiveness of acupuncture in smoking cessation	Drug Alcohol Rev	9	While acupuncture appears promising, there is insufficient evidence at this point, without further research, to recommend it as an effective form of therapy.
Baillie AJ 1994[52]	Meta-analytic review of the efficacy of smoking cessation interventions	Drug Alcohol Rev	5	In five studies of acupuncture compared with control, consistent results were found showing no benefit for acupuncture.
Di YM 2014[53]	A meta-analysis of Ear-acupuncture, ear-acupressure and auriculotherapy for cigarette smoking cessation	Drug Alcohol Depend	25	The Ear acupuncture/ear acupressure interventions appeared neither inferior nor superior to the behavioural interventions at 3 and 6 month follow-ups.
Kim SS 2012[54]	A systematic review of smoking cessation intervention studies in China	Nicotine Tob Res	3	TCM techniques such as acupuncture and ear point seed pressure yielded a much higher abstinence rate than pharmacotherapy and counseling.
Kitikannakorn N 2013[55]	An overview of the evidences of herbals for smoking cessation	Complement Ther Med	6	Evidence supporting the role of complementary medicines as an anti-smoking is limited.

Linde K 2001[56]	Systematic reviews of complementary therapies - an annotated bibliography. Part 1: acupuncture	BMC Complement Altern Med	4	Acupuncture is no more effective than sham acupuncture in smoking cessation.
White AR 1997[57]	Smoking cessation with acupuncture? A 'best evidence synthesis'	Forsch Komplementarmed	7	Acupuncture for smoking cessation does not produce an effect greater than placebo.
Cheng HM 2012[58]	Systematic review and meta-analysis of the effects of acupoint stimulation on smoking cessation	Am J Chin Med	16	A significant effect of acupoint stimulation was found in smoking cessation rates and cigarette consumption at immediate, 3- and 6-month follow-ups. Multi-modality treatments, especially acupuncture combined with smoking cessation education or other interventions, can help smokers to eschew smoking during treatment, and to avoid relapse after treatment.
White A 2006[59]	The effects of auricular acupuncture on smoking cessation may not depend on the point chosen–an exploratory meta-analysis	Acupunct Med	13	Auricular acupuncture appears to be effective for smoking cessation, but the effect may not depend on point location.
White AR 1999[60]	A meta-analysis of acupuncture techniques for smoking cessation(Review)	Tob Control	14	Acupuncture was not superior to sham acupuncture for smoking cessation; no particular aspect of acupuncture technique was associated with a positive effect.
White AR 2002[61]	Acupuncture for smoking cessation(Review)	Cochrane Database Syst Rev	22	The results with different acupuncture techniques do not show any one particular method (i.e. auricular acupuncture or non-auricular acupuncture) to be superior to control intervention.
White AR 2006[62]	Acupuncture and related interventions for smoking cessation(Review)	Cochrane Database Syst Rev	24	There is no consistent evidence that acupuncture, acupressure, laser therapy or electrostimulation are effective for smoking cessation, but methodological problems mean that no firm conclusions can be drawn. Further research using frequent or continuous stimulation is justified.

White AR 2011[63]	Acupuncture and related interventions for smoking cessation(Review)	Cochrane Database Syst Rev	33	There is no consistent, bias-free evidence that acupuncture, acupressure, laser therapy or electrostimulation are effective for smoking cessation, but lack of evidence and methodological problems mean that no firm conclusions can be drawn.
White AR 2014[46]	Acupuncture and related interventions for smoking cessation	Cochrane Database Syst Rev	38	Although pooled estimates suggest possible short-term effects there is no consistent, bias-free evidence that acupuncture, acupressure, or laser therapy have a sustained benefit on smoking cessation for six months or more.
